# Detection of *Streptococcus pneumoniae* and *Haemophilus influenzae* Type B by Real-Time PCR from Dried Blood Spot Samples among Children with Pneumonia: A Useful Approach for Developing Countries

**DOI:** 10.1371/journal.pone.0076970

**Published:** 2013-10-08

**Authors:** Laura Selva, Rachid Benmessaoud, Miguel Lanaspa, Imane Jroundi, Cinta Moraleda, Sozinho Acacio, Melania Iñigo, Alien Bastiani, Manuel Monsonis, Roman Pallares, Quique Bassat, Carmen Muñoz-Almagro

**Affiliations:** 1 University Hospital Sant Joan de Deu, Esplugues, Barcelona, Spain; 2 Barcelona Centre for International Health Research (CRESIB, Hospital Clínic-Universitat de Barcelona), Barcelona, Spain; 3 Centro de Investigação em Saúde de Manhiça (CISM), Maputo, Mozambique; 4 Fundació Clínic per a la Recerca Biomèdica, Rabat, Morocco; 5 Institut Nationald’Administration Sanitaire (INAS), Ministère de la santé, Rabat, Morocco; 6 Bellvitge Hospital, Idibell, Ciberes and University of Barcelona, Barcelona, Spain; Rockefeller University, United States of America

## Abstract

**Background:**

Dried blood spot (DBS) is a reliable blood collection method for storing samples at room temperature and easily transporting them. We have previously validated a Real-Time PCR for detection of *Streptococcus pneumoniae* in DBS. The objective of this study was to apply this methodology for the diagnosis of *S. pneumoniae* and Haemophilus influenzae b (Hib) in DBS samples of children with pneumonia admitted to two hospitals in Mozambique and Morocco.

**Methods:**

*Ply* and *wzg* genes of *S. pneumoniae* and *bexA* gene of Hib, were used as targets of Real-Time PCR. 329 DBS samples of children hospitalized with clinical diagnosis of pneumonia were tested.

**Results:**

Real-Time PCR in DBS allowed for a significant increase in microbiological diagnosis of *S. pneumoniae* and Hib. When performing blood bacterial culture, only ten isolates of *S. pneumoniae* and none of Hib were detected (3·0% positivity rate, IC95% 1·4-5·5%). Real-Time PCR from DBS samples increased the detection yield by 4x fold, as 30 *S. pneumoniae* and 11 Hib cases were detected (12·4% positivity rate, IC95% 9·0-16·5%; P<0·001).

**Conclusion:**

Real-Time PCR applied in DBS may be a valuable tool for improving diagnosis and surveillance of pneumonia caused by *S. pneumoniae* or Hib in developing countries.

## Introduction

Pneumonia is the main cause of death in children worldwide. It is estimated that it kills 1·2 million children under five years every year, accounting for 18% of all deaths in this population group [1]. *Streptococcus pneumoniae* and Haemophilus influenzae b (Hib) are the two principal causes of bacterial pneumonia [1], and also major causes of other invasive bacterial diseases. These pathogens can be prevented by immunization or treated with low cost antibiotics but it has been estimated that only 30% of children with bacterial pneumonia receive the antibiotics that they need [2].

The introduction of the Hib conjugate vaccine into national childhood immunization programs in the 1990s has resulted in a marked and sustained reduction in the incidence of invasive Hib disease in many countries [3-5]. Recently, this vaccine has been introduced in many developing countries through the Expanded Programs of immunization (EPI), with similar reported decreases in invasive Hib disease [6]. Similarly, in 2000 a conjugated vaccine against seven pneumococcal serotypes started to be implemented and showed to be highly effective to prevent pneumococcal disease caused by the serotypes included in the vaccine [7]. Since 2012, WHO recommends the introduction of two new pneumococcal conjugate vaccines (against 10 and 13 serotypes) in childhood immunization programs worldwide, since the additional serotype coverage represents an important progress against pneumococcal disease.

In order to monitor trends and detect changes in the epidemiology of *S. pneumoniae* and Hib after the introduction of the vaccines, routine surveillance of episodes caused by these pathogens is mandatory. While this is feasible in most developed settings, it is seldom performed in the developing world. Bacterial culture, traditionally considered the gold standard methodology for bacterial surveillance, is less sensitive than molecular methods in the diagnosis of pneumococcal and Hib invasive disease [8-10]. In the majority of developing countries, the prevalence of invasive disease caused by *S. pneumoniae* and/or Hib is not adequately known due to the scarcity of available laboratories, microbiological or molecular diagnostic tools and expertise. This hinders such countries’ surveillance efforts, and thus their potential application to international funds to support the introduction of conjugate vaccines. Moreover, sample preservation prior to shipment to national/international reference laboratories poses an additional challenge and may jeopardize the quality of collected material [11].

Dried blood spot (DBS) is a reliable method of blood collection used for the diagnosis of several human diseases. DBS is a particularly useful method for storing samples and diagnosing pediatric conditions in which often very small volumes of sample are available, and for the screening of high-risk populations especially in countries where health care facilities are not readily accessible [12,13]. We have previously described that the DBS technique enables reproducible storage of samples for identification and serotyping of *S. pneumoniae* and that its use is an attractive method for preserving samples at room temperature and easily transporting them [14].

The main objective of the present study was to evaluate the presence of *S pneumoniae* and Hib in DBS samples from pediatric patients with diagnosis of clinical pneumonia in two distinct epidemiological settings in Africa: Manhiça District Hospital (MDH), Mozambique; and the *Hôpitald’enfants de Rabat* (HER), Morocco. DBS samples of a control group of healthy children recruited in MDH were also analyzed.

## Materials and Methods

### Study design and characteristics of patients and healthy controls included in the study

DBS samples were obtained from children participating in two prospective studies on the epidemiology of acute respiratory infections conducted in Mozambique and Morocco. In Manhiça, Mozambique, children younger than 10 years admitted to MDH with a clinical diagnosis of pneumonia according to WHO criteria [15] from February 2010 to June 2012 were recruited. In Rabat, Morocco, recruitment covered children aged 2 months to 5 years admitted to the HER with the same clinical diagnosis from January to December 2011. WHO clinical pneumonia criteria include the presence of an increased respiratory rate (according to age), cough and/or difficulty in breathing [15]. These patients were recruited by researchers from the Barcelona Center of International Health Research (CRESIB) under informed consent as part of routine bacteriological surveillance of pneumonia. According to study procedures, a conventional blood culture was performed for all patients enrolled in the study at both sites, together with a chest x-ray and a nasopharyngeal sample collection for viral determination. Blood cultures were processed in the microbiology laboratory of the Manhiça Health Research Center (CISM) adjacent to the MDH and in the research laboratory of the HER, which both operate under similar standard operating procedures. In brief, one to 3 ml of whole blood were inoculated in a pediatric blood culture bottle (Pedibact®, Becton-Dickinson, Franklin Lanes, NJ, USA) and incubated in an automated system (BACTEC® 9050, Becton-Dickinson) for a minimum of 4 days. Positive blood cultures were examined by Gram stain and subcultured on blood, chocolate or MacConkey agar plates. Bacterial isolates were identified by colony biochemical conventional methods, colony morphologic analysis and growth requirements. Additionally, four drops of blood were impregnated on properly identified DBS, dried at room temperature and later inserted into individual envelopes for shipment to University Hospital Sant Joan de Déu in Barcelona.

DBS were also obtained as a control procedure from healthy community children aged between 0-10 years, randomly recruited from the Manhiça area, using the ongoing demographic surveillance system and census run by CISM. Parents or guardians of patients were also requested to provide a written informed consent prior to inclusion in the study. Children with respiratory illness requiring hospitalization or medical treatment were excluded as controls. Children with mild respiratory symptoms (e.g. cough, blocked nose or runny nose) were not excluded. However, information about any current symptoms was collected via a standardized questionnaire. DBS from controls-children and DBS from case-patients were shipped together to Barcelona.

Hib vaccine was introduced in Morocco in 2007, and is currently included in its expanded Programme of Immunization (EPI). Mozambique is an eligible country of the GAVI Alliance and has been assisted in the areas of immunization and health development since 2001. In April 2009 Hib vaccine was introduced countrywide through the Mozambique’s EPI. No pneumococcal conjugate vaccines were introduced in Mozambique and Morocco during the study period. The burden of Hib invasive disease in Mozambique is well documented and high [16] while very scarce data on its incidence or impact are available in Morocco [17].

### Ethics Statement

The study protocols were both approved by the corresponding National Ethics Committees (*Comité Nacional de Bioética em Saúde de Moçambique*, CNBS, Maputo, Mozambique; and *Comité d’Éthique de la recherché Biomédicale*, (Départ N° 1252-16 Déc 2009) of the Faculty of medicine in Rabat, Morocco) together with the Hospital Clínic of Barcelona Ethics Review Committee. Patients were recruited by researchers from the Barcelona Center of International Health Research (CRESIB) under informed consent. Parents or guardians of patients were also requested to provide a written informed consent prior to inclusion in the study.

### Hib DNA detection by Real-Time PCR in DBS *samples*

Real-Time PCR for *S. pneumoniae* and Hib were performed in all DBS (of patients and healthy controls). DNA extraction was performed through the NucliSENS® easyMAG^TM^(BioMérieux) system according to manufacturer’s instructions and using a protocol that includes an external lysis step.

Detection of *S. pneumoniae* was carried out by published procedures that included the study of pneumolysin (*ply*) and *wzg* genes (both had to be simultaneously positive to confirm any case as a positive pneumococcal infection), and subsequent capsular typing of *S. pneumoniae* DNA positive samples [14,18]. Real-Time PCR of Hib was based on the detection of *bexA* gene [19]. The Hib Real-Time PCR performed with DBS was validated following the previously described methodology [14]. Internal controls for monitoring false negatives by PCR inhibitors were run for each sample to monitor the overall assay performance and these consisted of 1 µl of TaqManRnase P control reagent (VIC) (Applied Biosystem, Foster City, CA) that included human RNAse P primers and probe. Negative results were defined as those with cycle threshold (Ct) values above 40. Invalid results were defined as those which showed no amplification of the target gene and the internal control.

### Statistical Analysis

Statistical analyses were performed using SPSS Statistics for Windows software (version 19·0, IBM Corp.). Continuous variables were compared using the t-test or the Mann-Whitney U test, and categorical variables were compared using the Chi-square test or Fisher exact test, as required. Confidence Intervals (CI) were set at 95% and significance at a 2-sided p-value of <0·05 for all statistical analyses.

## Results

During the study period 329 patients fulfilling the inclusion criteria with clinical diagnosis of pneumonia and 104 healthy controls had DBS samples collected. Table 1 shows the demographic characteristics of patients and controls included in the study.

**Table 1:** Patients and control group characteristics.

Characteristics	All	MDH (%)	HER (%)
**Patients**	329	231	98
Sex			
Male	174	112 (48.5)	62 (63.2)
Female	155	119 (51.5)	36 (36.8)
Age in months, median (range)	16 (0-119)	13 (0-119)	23 (0-78)
Age group			
<24 months	213	163 (70.6)	50 (51.0)
24-59 months	98	54 (23.4)	49 (49.0)
>59 months	14	14 (6)	
**Control group**	104	104	
Sex			*
Male	45	45 (43.3)	*
Female	59	59 (56.7)	*
Age in months, median (range)	37 (3-117)	37 (3-117)	*
Age group			
<24 months	25	25 (24)	*
24-59 months	63	63 (60.6)	*
>59 months	16	16 (15.4)	*

MDH: Manhiça District Hospital; HER: *Hôpitald’enfants de Rabat.* * No patients available in HER

Blood culture samples were collected from all 329 patients, 17 of which (5·2%) were positive and 16 isolates (4·9%) were considered contaminants. Among pathogenic bacteria, *Streptococcus pneumoniae* was the most frequently isolated pathogen (n=10; 3·1%) followed by *Staphylococcus aureus* (n=2; 0·6%) and *Salmonella* spp (n=2; 0·6%). No Hib isolates were detected in any of the two sites through the use of conventional blood culture (Table 2).

**Table 2:** Distribution of isolates by bacterial culture among children of Mozambique and Morocco.

Pathogen		Positive	%
*Streptococcus pneumoniae*		10	3.1
*Staphylococcus aureus*		2	0.6
*Salmonella* spp		2	0.6
*Pseudomonas* spp		1	0.3
*Burkholderia cepacia*		1	0.3
Other BGN		1	0.3
Potential contaminants			
Coagulase negative *Staphylococcus*		12	3.6
*Micrococcus luteus*		1	0.3
Gram positive bacillus		3	0.9
Negative		296	89.9
Total pathogens detected		17*	5.1

* Only considering clinically significant isolates

Real-Time PCR was performed in 329 DBS samples for Hib study and in 254 for *S. pneumoniae*. DNA extracts of first series of 75 DBS consecutive samples of MDH were not available for the study of *S. pneumoniae*, because samples had been fully utilized for other molecular studies (data not shown). Among these 75 cases, isolation of *S. pneumoniae* by blood culture was detected in four patients and the rest were negative. Detection of DNA of *S. pneumoniae* in DBS was found in 23 (9%) of 254 patients studied (median age 16 months, range 4-93 months). 18 patients were attended in MDH and 5 in HER; 3 additional patients with isolation of *S. pneumoniae* by culture were not detected by PCR. It is noteworthy that 20 patients (87%) had a positive DBS result with a negative blood culture for *S. pneumoniae*.

Hib DNA was detected by Real-Time PCR in 11 (3·3%) DBS samples of 329 patients (median age 11 months, range 2-38 months). A negative result was obtained in 311 DBS samples (94·5%) and an invalid result in 7 (2·1%). The proportion of positive samples was higher in MDH (9 of 231; 3·9%) than in HER (2 of 98; 2%) but without significant differences (p=0·19). Among 11 DBS with positive detection of Hib DNA, 10 of them were negative by blood culture and one yielded positive for *S. pneumoniae.*

The addition of Real-Time PCR in DBS allowed a significant increase in the detection capacity of microbiological diagnosis of *S. pneumoniae* and Hib in the two health centers and in all groups of age (Table 3). Indeed, with blood cultures, only ten isolates of *S. pneumoniae* and none of Hib were detected (3·0% positivity rate, IC95% 1·4-5·5%), while by adding Real-Time PCR,30 *S. pneumoniae* and 11 Hib were identified; meaning a net four-fold increase in positivity rate (n=41 cases, 12·4% positivity rate, IC95% 9·0-16·5%; P<0·002).

**Table 3:** Microbiological diagnosis of patients according to age group and geographical site.

Age group		Patients studied	Patients diagnosed by culture *n* (%; 95%CI)	Patients diagnosed only by real-time PCR *n*(%; 95%CI)	Total patients diagnosed *n* (%; 95%CI)
Manhiça District Hospital (MDH)					
*S. pneumoniae*					
<24 months		109	3 (2.75; 0.6-8.1)	11 (10.1; 5.6-17.3)	14 (12.9; 7.6-20.5)
24-59 months		33	2 (6.1; 0.7-20.6)	5 (15.1; 6.1-31.4)	7 (21.2; 10.4-38.1)
>59 months		14	2 (14.3; 2.8-41.1)	2 (14.3; 2.8-41.1)	4 (28.6; 11.3-55)
Overall		156	7 (4.5; 2-9.1)	18 (11.5; 7.3-17.6)	25 (16; 11-22.6)
*H. influenzae* B					
<24 months		163	0	6 (3.7; 1.5-8)	6 (3.7; 1.5-8)
24-59 months		54	0	3 (5.6; 1.3-15.7)	3 (5.6; 1.3-15.7)
>59 months		14	0	0	0
Overall		231	0	9 (3.9; 1.9-7.3)	9 (3.9; 1.9-7.3)
*Hôpitald’enfants de Rabat* (HER)					
*S. pneumoniae*					
<24 months		50	0	3 (6; 1.4-16.8)	3 (6; 1.4-16.8)
24-59 months		48	0	2 (4.5; 0.4-16)	2 (4.5; 0.4-16)
Overall		98	0	5 (5.1; 1.9-11.7)	5 (5.1; 1.9-11.7)
*H. influenzae* B					
<24 months		50	0	2 (4; 0.3-14.2)	2 (4; 0.3-14.2)
24-59 months		44	0	0	0
>59 months		4	0	0	0
Overall		98	0	2 (2; 0.1-7.5)	2 (2; 0.1-7.5)

Among the 104 healthy community controls all DBS were negative by Hib Real-Time PCR and 2 were positive for *S. pneumoniae*. These corresponded to two children of 24 and 54 months of age both of whom presented mild respiratory symptoms. Therefore Hib Real-Time PCR seemed more specific (100%) than *S. pneumoniae* (98%) but without significant differences (P=0·5). The lack of culture results for the community controls however does not allow us to confirm whether such patients were false or true positives.

## Discussion

We had previously validated the use of DBS for diagnosis of *S. pneumoniae* by using molecular methods [14]. In this study, a Real-Time PCR for the analysis of Hib was developed and adapted for use with DBS samples providing good sensitivity. Dried blood spot (DBS) is a useful and easy-to-use method for preserving and transporting blood samples which may have a particular utility in developing countries, where microbiology laboratories are rare and technical expertise is limited. DBS blood collection provides a long-lasting and temperature-stable method for storage of biological samples, and therefore may be suitable for the diagnosis of bacterial or viral infections by Real-Time PCR, as other authors have described [12,14,20,21].

Although introduction of the Hib vaccine has resulted in a reduction of the disease caused by Hib in Morocco [22], this pathogen can still be detected as a cause of bacterial pneumonia when we perform Real-Time PCR in DBS. The results presented here, together with the reported burden of *S. pneumoniae* and Hib [23] show that these pathogens continued to cause more than 50% of microbiological confirmed severe clinical pneumonia episodes. These data highlight the need to support widespread implementation of the demonstrated highly effective conjugate vaccines for these two pathogens. A potential limitation in the Hib PCR applied in this study is that bexA primers not only amplify Hib but also *H. influenzae C* (Hic). However, as Hic is a very uncommon cause of invasive disease in children we do not believe it could importantly affect our results.

Similarly to other previous evaluations, the performance of classical microbiology to detect bacterial pathogens was poor, detecting only a bacterial pathogen in a mere 5·2% of the episodes. It is noteworthy that, Hib was not isolated in any of the studied children by blood culture. Although some of these cases may have been caused by non-bacterial pathogens or conditions, the clinical manifestations and chest X-ray characteristics of these patients were highly suggestive of a high burden of bacterial-associated disease (data not shown). The low positive rate of blood culture is similar to that reported by other authors in pediatric populations, both for developed and developing countries [24,25]. Notably, with the addition of two specific Real-Time PCR for Hib and *S. pneumoniae* a four-fold significant increase of the detection yield was achieved. If we had included other molecular tests to improve detection of pathogens such as *Mycoplasma pneumoniae*, *S.* aureus*, Salmonella* or *Klebsiella*, among others, the increase of etiological diagnosis may have been even more dramatic.

The lack of *H. influenzae* isolates detected using bacterial cultures may be explained on account of *Haemophilus* *spp* being fastidious organisms, difficult to grow in usual culture conditions. Moreover, isolation rates can be further reduced when samples are not adequately processed prior to their arrival to the laboratory [26,27]. The use of antibiotics before obtaining the sample, or the limited amount of blood that can be obtained from young children are two other important factors limiting the performance and decreasing the sensitivity of traditional blood cultures, not only for the detection of Hib but also for other pathogens. Considering that children aged less than 2 years are the age group at a higher risk of Hib invasive disease [28], a fact supported by our data (median age was 15·3 months) and that the amount of blood that can be obtained from these children seldom exceeds 1-2mL it is understandable that detection yields of blood culture are necessarily low, when compared to the volumes that can be used in older children or adults (5-10 ml). Microbiological diagnosis by Real-Time PCR in only 1-2 drops of blood impregnated in a paper spot is a really interesting contribution to the available diagnostic tools that can bypass some of the inherent problems of bacterial culture. Moreover, we can report that the technique is highly specific, as data among healthy control show and also proves to be sensitive, as shown by the significant increase of microbiological diagnosis when PCR was added.

However, three children with isolation of *S. pneumoniae* by culture were not detected by PCR, probably due to the low bacterial load and the minimum quantity of sample processed.

In this study we found a co-infection between *S. pneumoniae* and Hib. Recently, van der Bergh et al. [29] have reported an interesting study that shows *S. pneumoniae* colonization is positively associated with the presence of *H. influenzae*. In addition other authors have reported co-infection of *S. pneumoniae* and Hib in children with otitis media [30]. However little is known about co-infection of these pathogens in blood of patients with pneumonia, probably due to the low sensitivity of current microbiological methods.

In conclusion, in countries where health services are not easily accessible, and where scarce epidemiological surveillance systems exist for infectious diseases, molecular testing applied to DBS can overcome the obstacles associated with the collection, storage and shipment of samples, facilitating the detection of *S. pneumoniae* and Hib. Thus, improving the etiological surveillance of pneumonia in countries with poor resources may have critical implications for prevention and treatment policies. The promising results published in the present study should encourage exploring the detection of other pathogens with the same strategy.
