# Correction: Detection of *Streptococcus pneumoniae* and *Haemophilus influenzae* Type B by Real-Time PCR from Dried Blood Spot Samples among Children with Pneumonia: A Useful Approach for Developing Countries

**DOI:** 10.1371/journal.pone.0147678

**Published:** 2016-01-19

**Authors:** Laura Selva, Rachid Benmessaoud, Miguel Lanaspa, Imane Jroundi, Cinta Moraleda, Sozinho Acacio, Melania Iñigo, Alien Bastiani, Manuel Monsonis, Roman Pallares, Quique Bassat, Carmen Muñoz-Almagro

The following information is missing from the Funding section of the published article:

This work is funded by the "Plan Nacional de I+D+I and Instituto de Salud Carlos III- Subdirección General de Evaluación y Fomento de la Investigación Sanitaria", project PI10/02058, and the European Regional Development Fund (FEDER).
